# *Coxiella burnetii* in Ticks, Argentina

**DOI:** 10.3201/eid1902.120362

**Published:** 2013-02

**Authors:** Richard C. Pacheco, Ignacio E. Echaide, Rosiane N. Alves, Marcelo E. Beletti, Santiago Nava, Marcelo B. Labruna

**Affiliations:** Author affiliations: Federal University of Mato Grosso, Cuiabá, Brazil (R.C. Pacheco);; Instituto Nacional de Tecnología Agropecuaria, Rafaela, Argentina (I.E. Echaide, S. Nava);; Federal University of Uberlândia, Uberlândia, Brazil (R.N. Alves, M.E. Beletti);; University of São Paulo, São Paulo, Brazil (M.B. Labruna)

**Keywords:** *Coxiella burnetii*, *Amblyomma*, ticks, isolation, Argentina, vector-borne diseases, bacteria

**To the Editor:** The Gammaproteobacterium *Coxiella burnetii* is the causative agent of acute Q fever and chronic endocarditis in humans worldwide. It is transmitted primarily by aerosol route or by ingestion of fomites from infected animals, mostly from domestic ruminants (*1*). Although >40 tick species can be infected with *C. burnetii,* direct transmission of this agent to humans from infected ticks has never been properly documented. However, ticks may play a critical role in the transmission of *C. burnetii* among wild vertebrates (*1*). Only a few studies, mostly related to human clinical cases or seroepidemiogic evaluation of healthy animals, have reported *C. burnetii* in South America (*2*–*4*). However, to our knowledge, *C. burnetii* has never been reported in ticks in the continent.

During ecologic studies on *Amblyomma parvum* and *A. tigrinum* ticks in the Córdoba Province of Argentina, engorged nymphs were collected from the common yellow toothed cavy (the rodent *Galea musteloides*) (*5*,*6*). In the laboratory, engorged nymphs molted to adults (92 *A*. *tigrinum*, 13 *A. parvum*), which were individually submitted to the hemolymph test with Gimenez staining for detection of rickettsiae-like organisms (*7*). By the hemolymph test, 1 *A. tigrinum* female, and 2 *A. parvum* male ticks were found to contain red-stained rickettsiae*-*like structures. These 3 ticks were processed individually by the shell vial technique, with the purpose of isolating intracellular bacteria in Vero cell culture (*7*). Inoculated cells were always incubated at 28°C. Intracellular bacteria were successfully isolated from all 3 ticks and established in Vero cell culture, as demonstrated by Gimenez staining of infected cells from at least 10 subsequent passages, which all infected 100% of the cells (Figure, panel A). Infected Vero cells contained multiple vacuoles (Figure, panel B) that enclosed a seething mass of microorganisms (Video), compatible with *Coxiella* organisms. Such vacuoles were not seen in uninfected control Vero cells incubated under the same conditions as those of infected cells (Figure, panel C).

**Figure:**
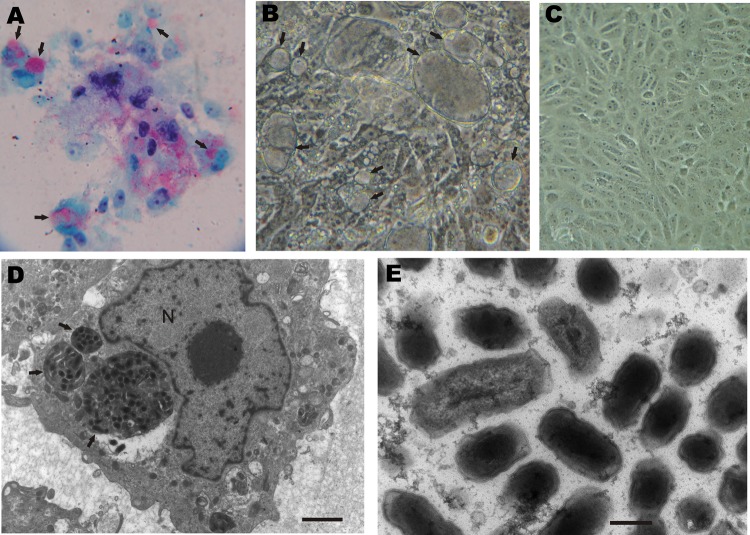
Vero cells inoculated with *Amblyomma* tick extracts for isolation of rickettsiae. A) Rickettsiae-like organisms stained in red by Gimenez staining (original magnification ×400). B) Inoculated monolayer photographed under phase-contrast microscopy (original magnification ×400). C) Uninfected control monolayer under phase-contrast microscopy (original magnification ×400). D) Transmission electron microscopy of infected cells. E) Transmission electron microscopy image of intravacuolar bacteria. Bar indicates 250 nm. N, nucleolus. Arrows indicate vacuoles containing bacteria.

**Video:**
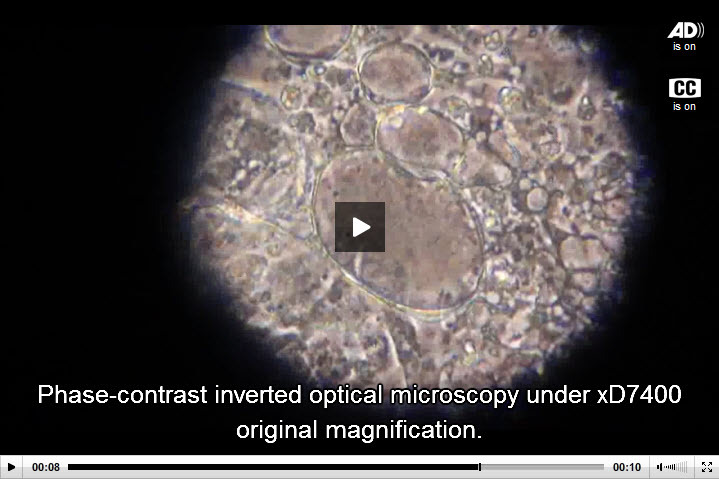
Vero cells infected by *Coxiella burnetii* isolated from an *Amblyomma parvum* tick. Note large vacuoles enclosing a seething mass of microorganisms. Phase-contrast inverted optical microscopy under ×400 original magnification.

Direct Video Link: http://streaming.cdc.gov/vod.php?id=1a1ecc70615f6b603c323f9620fecb6720121212092235186

For molecular analyses, DNA from the infected cells of each of the 3 isolates was extracted by boiling at 100°C for 10 min; it yielded products of the expected size through PCR protocols selective for portions of 3 genes of the genus *Coxiella*: primers QR-FO (5′-ATTGAAGAGTTTGATTCTGG-3′) and QR-RO (5′-CGGCCTCCCGAAGGTTAG-3′) for the 16S rRNA gene (*8*); primers CAPI844F (5-ATTTAGTGGGTTTCGCGCAT-3′) and CAPI844R (5′-CATCAGCATACGTTTCGGGAA-3′) for the *cap* gene (*9*); and primers Cox-F-pry2 (5′-TTATTTACCAACGTTCCTGAGCCG-3′) and Cox-R-pry2 (5′-TTTATCCCGAGCAAATTCAATTATGG-3′) for the *pyrG* gene (*9*). PCR products underwent DNA sequencing in an automatic sequencer (Applied Biosystems/PerkinElmer, Foster City, CA, USA) according to the manufacturer`s protocol. We sequenced 1,386, 557, and 545 nt of the genes 16S rRNA, *cap*, and *pyrG*, respectively, which were identical to each other for each gene amplified from the 3 tick isolates. By BLAST analyses (www.ncbi.nlm.nih.gov/blast), these sequences were 99.9% (1,384/1,386 nt), 99.6% (556/558 nt), and 99.6% (452/454 nt) identical to the corresponding GenBank sequences of the North American *C. burnetii* genes 16S rRNA, *cap*, and *pyrG*, respectively (HM208383, CP001020, CP001020). Partial sequences (16S rRNA, *cap*, *pyrG*) from *C. burnetii* generated in this study were deposited into GenBank and assigned nucleotide accession nos. JQ740886–JQ740888, respectively.

Infected Vero cell monolayers were fixed in a modified Karnovsky solution, stained with uranyl acetate and lead citrate, and examined in a transmission electron microscope according to standard procedures. Ultrastructurally, *Coxiella* organisms were identified by morphologic features within heavily infected Vero cells. The organisms possessed typical bacillary morphologic characteristics and were observed inside vacuoles (phagolysosomes) of different sizes, proportional to the number of organisms (Figure, panels D, E). Intravacuolar organisms had a mean length of 0.55 ± 0.13 µm (range 0.42–0.85 µm) and a mean width of 0.25 ± 0.03 µm (range 0.22–0.32 µm).

Ticks negative for rickettsiae-like organisms by hemolymph testing were subjected individually to DNA extraction by the guanidine isothiocyanate-phenol technique (*10*) and screened for *Coxiella* spp. by PCR that targeted the *pyrG* gene, as described above. Although no *A. parvum* tick yielded amplicons, 40 *A. tigrinum* ticks yielded amplicons of the expected size for the *pyrG* gene. DNA sequences generated from these ticks were identical to the *pyrG* partial sequences obtained from the *C. burnetii* isolates mentioned above.

We found 41 (44.6%) of 92 ticks and 2 (15.4%) of 13 of the *A. tigrinum* and *A. parvum* adult ticks, respectively, to be infected by *C. burnetii.* Because these ticks were collected as engorged nymphs from wild rodents in a natural biome of Argentina, namely, the Chaco phytogeographic domain (*5*,*6*), our results indicate that *C. burnetii* is established in this part of the country where ticks possibly play an essential role in the enzootic cycle. Serologic evidence of *C. burnetii* infection has been found among goats and cattle in several areas of Argentina (*3**,**4*). Because free-ranging domestic cattle and goats are considered among the most likely hosts for *A. parvum* adult ticks in the Chaco domain (*6*), humans are likely being exposed to *C. burnetii* as well. 
